# Silymarin suppresses basal and stimulus-induced activation, exhaustion, differentiation, and inflammatory markers in primary human immune cells

**DOI:** 10.1371/journal.pone.0171139

**Published:** 2017-02-03

**Authors:** Erica S. Lovelace, Nicholas J. Maurice, Hannah W. Miller, Chloe K. Slichter, Robert Harrington, Amalia Magaret, Martin Prlic, Stephen De Rosa, Stephen J. Polyak

**Affiliations:** 1 Department of Laboratory Medicine, University of Washington, Seattle, WA, United States of America; 2 Vaccine and Infectious Disease Division, Fred Hutchinson Cancer Research Center, Seattle, WA, United States of America; 3 Department of Global Health, University of Washington, Seattle, WA, United States of America; 4 Division of Allergy and Infectious Disease, University of Washington, Seattle, WA, United States of America; 5 Department of Biostatistics, University of Washington, Seattle, WA, United States of America; 6 Department of Microbiology, University of Washington, Seattle, WA, United States of America; Jackson Laboratory, UNITED STATES

## Abstract

Silymarin (SM), and its flavonolignan components, alter cellular metabolism and inhibit inflammatory status in human liver and T cell lines. In this study, we hypothesized that SM suppresses both acute and chronic immune activation (CIA), including in the context of HIV infection. SM treatment suppressed the expression of T cell activation and exhaustion markers on CD4+ and CD8+ T cells from chronically-infected, HIV-positive subjects. SM also showed a trend towards modifying CD4+ T cell memory subsets from HIV+ subjects. In the HIV-negative setting, SM treatment showed trends towards suppressing pro-inflammatory cytokines from non-activated and pathogen-associated molecular pattern (PAMP)-activated primary human monocytes, and non-activated and cytokine- and T cell receptor (TCR)-activated mucosal-associated invariant T (MAIT) cells. The data suggest that SM elicits broad anti-inflammatory and immunoregulatory activity in primary human immune cells. By using novel compounds to alter cellular inflammatory status, it may be possible to regulate inflammation in both non-disease and disease states.

## Introduction

Inflammation is a protective and reparative response that is induced by pathogen or host-derived engagement of pattern recognition receptors (PRR) as well as through the engagement of cytokine and non-cytokine cellular receptors [1, 2]. Receptor activation triggers cellular signal transduction, causing production and release of pro-inflammatory cytokines and chemokines from cells, which in turn, recruits immune effector cells to the site of inflammation. Upon resolution of infection and/or damage, inflammatory responses normally return to baseline. Human immune cells are on the front line of many inflammatory responses, and include CD4+ and CD8+ T cells, monocytes, and mucosal associated invariant T (MAIT) cells. Temporally, monocytes and MAIT cells comprise the initial innate phase of an inflammatory response, while CD4+ and CD8+ comprise the adaptive phase and require proper inflammatory cues (from MAIT cells or monocytes) for their effector function, the quality of the immune response, and formation of a memory population. Dysregulated inflammation interrupts this regimented, temporal process.

In the case of persistent infections, dysregulated inflammation is maintained, establishing a state of chronic immune activation (CIA), which can lead to various disease states. Chronic HIV infection, despite the effective control of viremia with antiretroviral therapy (ART), is a state of CIA that leads to a host of inflammatory disorders in many infected patients [3]. With CIA, memory T cell effector functions are lost, inhibitory factors are induced, and immune cell metabolism is altered [4]. In both ART-treated and untreated HIV-infected individuals, CIA is associated with significantly elevated immune activation markers [5], various inflammatory diseases [6], cardiovascular diseases [7], both AIDS-defining and non-AIDS defining cancers [8], as well as HIV disease progression and mortality [9].

CIA in the context of HIV infection may be due to several factors [10] and can be assessed by measuring exhaustion or proliferation markers on immune cells [11], changes in immune cell inflammatory function [12], and the loss of the CD4+ T-cell population causing in an inverted CD4+/CD8+ ratio [13]. For example, the activation marker, CD38, on CD8+ and CD4+ T cells, is considered one of the best correlates for disease progression [14]. Programmed cell death protein (PD-1), which is highly expressed on exhausted T cells, is also upregulated in T cells in HIV-infected persons [15]. As such, various approaches have been used to reduce CIA including direct blockade of cellular exhaustion markers, such as targeting PD-1 and cytotoxic T lymphocyte antigen 4 (CTLA4) [16]. In addition to targeting exhaustion markers, dysregulated inflammation has also been shown to be suppressed with anti-inflammatory drugs such as aspirin [17], chloroquine [18], prednisone [6], and statins [19], all of which have been shown to reduce some parameters of CIA.

Silymarin (SM) is an herbal extract derived from the seeds of the milk thistle plant *Silybum marianum* [L.] Gaertn. [Asteraceae] and is frequently consumed by HCV- and HIV-infected subjects [20]. SM is known to suppress HCV infection *in vitro* [21–25] while an intravenous formulation of silibinin (a major component of SM) inhibits HCV replication *in vivo* [26–29], and inhibits HIV-1 infection *in vitro* [30]. In addition to its antiviral activities, SM suppresses various inflammation pathways: including inhibition of pro-inflammatory signaling pathways (e.g., NF-κB and forkhead box O [FOXO]), and the expression of pro-inflammatory cytokines and chemokines (e.g., CXCL1, CXCL2, CXCL8, CXCL10, IL-1, TNF-α [21, 22, 31, 32]. Furthermore, SM treatment blocks T cell activation [21, 22, 24, 33] and PHA-induced activation of peripheral blood mononuclear cells (PBMC) *in vitro* [30].

In this study, we explored the *in vitro* anti-inflammatory and immunomodulatory activities of SM in different primary human immune cells and contexts, including monocytes, MAIT cells, and T cells from HIV-infected and non-infected subjects.

## Materials and methods

### Silymarin preparation

Powdered extract (Product No. 345066, Lot No. 286061) of the seeds (achenes) of *Silybum marianum* [L.] Gaertn. was obtained from Euromed, S.A. (Barcelona, Spain), which is a part of the Madaus Group (Cologne, Germany). To eliminate stability concerns with freeze-thawing solutions of SM and the hygroscopic nature of DMSO, single use aliquots of SM were prepared as described [34, 35]. SM was reconstituted to a concentration of 10 mM in MeOH (based on a molecular weight of 482 g/mol for the seven main flavonolignan diastereoisomers). Then, 100 μL of this solution was dispensed into 0.7 mL microcentrifuge tubes and allowed to freeze-dry overnight, imparting 0.482 mg of SM per tube. The dried aliquots were stored at -20°C. Single-use aliquots of silymarin were reconstituted in 40 μL of DMSO and extensively vortexed to generate a 25 mM stock solution. DMSO solvent controls ([DMSO] ≤ 0.3%) were used for all experiments.

### Monocyte and MAIT cell isolation and culture

Human monocytes were isolated from cryopreserved PBMCs from HIV-negative subjects using MACS CD14 Microbeads (Miltenyi Biotec, Bergisch Gladbach, Germany). CD14+ monocytes were rested or stimulated with LPS, a TLR4 agonist, or ssRNA, a TLR8 agonist, and then treated with SM or DMSO for 24 hours, followed by supernatant collection, and then analyzed by Luminex. Following the CD14 microbead isolation of monocytes, the remaining cells were stained with antibodies against CD161 and Va7.2 and MAIT cells were isolated by FACS [29]. MAIT cells were rested, stimulated with IL-12/15/18 (100ng/ml each) or stimulated with anti-CD3/CD28 beads (Dynabeads, Invitrogen) + IL-12/15/18 as described [36]. Rested and stimulated MAIT cells were incubated with or without SM. Cellular phenotypes were assessed based on marker expression (measured by flow cytometry) and cellular function was based on cytokine production (measured by Intracellular Cytokine Stain [ICS] assay).

### PBMC samples and culturing

PBMC samples from HIV-infected subjects were obtained from the University of Washington (UW) Center for AIDS Research (CFAR) Specimen Repository. All PBMC specimens are linked to comprehensive clinical data within the UW HIV Information System allowing the identification of patients who HIV-positive and receiving ART, or HIV-positive and ART-naïve or not on ART at the time of donation. Inclusion and exclusion criteria and subject characteristics are listed in **Tables 1** and **2**.

**Table 1:** Inclusion and Exclusion Criteria for selecting PBMC samples.

Inclusion Criteria	Exclusion Criteria
• Men and women who are 18 years of age and older. • HIV-1 plasma RNA <50 copies/ml for at least three years with at least two viral load measures per year, and the most recent viral load within three months of screening. • Episodes of a single HIV plasma RNA 50–199 copies/ml will not exclude participation if the subsequent HIV plasma RNA was <50 copies/ml. • Receiving combination antiretroviral therapy (at least three agents). • In the last six months have two CD4+ T-cell counts greater than 500 cells/μl. • Documented subtype B HIV infection. • Able to give informed consent.	• Any significant acute medical illness in the past 8 weeks. • Any evidence of an active AIDS-defining opportunistic infection. • Active alcohol or substance use.Moderate to severe hepatic impairment. Hepatic transaminases (AST or ALT) > 3 x upper limit of normal (ULN). • Chronic hepatitis C. Hepatitis B infection as indicated by the presence of Hepatitis B surface antigen or detectable DNA levels in blood. Receipt of immunomodulating agents, immunization or systemic chemotherapeutic agents within 28 days prior to screening. • Receipt of histone deacetylase inhibitors at any time, and use of any of the following within 90 days prior to entry: systemic cytotoxic chemotherapy; investigational agents; immunomodulators (colony-stimulating factors, growth factors, systemic corticosteroids, rapamycin-like drugs, HIV vaccines, immune globulin, interleukins, interferons); Coumadin, warfarin, or other Coumadin-derived anticoagulants.

**Table 2:** Characteristics of Subjects Whose PBMCs Were Analyzed by Flow Cytometry.

Clinical Parameter		Median	IQR*
ART Status			
	HIV- (n = 4)	N/A	N/A
	HIV+ on ART (n = 14)	N/A	N/A
	HIV+ART-naïve (n = 7)	N/A	N/A
Age			
	<50 years (n = 13)	38	32.5–46
	≥50 years (n = 12)	54	51.3–56.8
CD4:8 Ratio			
	HIV- (n = 4)	N/A	N/A
	Good (≥ 1) (n = 6)	1.5	1.2–1.8
	Poor (< 1) (n = 15)	0.3	0.2–0.5

*IQR, interquartile range.

HIV+ cryopreserved PBMC were thawed in a 37°C water bath, transferred to a 50 ml conical tube containing 10 ml of R10 medium (RPMI 1640 with 25 mM HEPES buffer and L-glutamate [Gibco BRL Life Technologies, Waltham, MA, USA; 10% fetal bovine serum [FBS; Gemini Bio-products, West Sacramento, CA, USA], and Penicillin-Streptomcyin [Gibco BRL Life Technologies, Waltham, MA, USA]), centrifuged at 250 x g for 10 minutes, and the cell pellet was resuspended in R10 medium. Cell count and viability were determined using a Guava Viacount (EMD Millipore, Billerica, MA, USA), resuspended to approximately 2 x 10^6^ cells/ml in PBS (Gibco BRL Life Technologies, Waltham, MA, USA), and stained for flow cytometry analysis *ex vivo* or prepared for culture including treatment with SM (at 80 μM) or DMSO (as a vehicle control) for 72 hours at 37°C. After 72 hours in culture, cell counts and viability were again measured prior to marker staining and flow cytometry. Thirty-three samples were initially evaluated, and eight samples were excluded because of low viability. Thus, statistical analyses are based on samples from 25 subjects.

### Eleven-color polychromatic flow cytometry

Using an 11-marker flow cytometry antibody panel (listed in **Table 3**), we assessed CD4+ and CD8+ T cell phenotypes by measuring the expression of activation and exhaustion markers in HIV+ PBMC. We adopted our protocol as described previously [37]. Samples were stained with Aqua Live/Dead Fixable Dead Cell Stain (AViD; [38]) then surface-stained with antibodies against CD25, CD38, CD127, CTLA4, HLA-DR, and PD1. Cells were then fixed and permeabilized using FIX & PERM Cell Fixation and Cell Permeabilization Kit (ThermoFisher Scientific, Waltham, MA, USA). Cells were stored at -80°C in the dark overnight in FACS Wash (PBS with 2% FBS) following permeabilization. The following day, cells were thawed at 37°C for 10 minutes, and then intracellular staining was performed with antibody against CD3, CD4, CD8, and Ki67. Following intracellular staining, cells were stored at 4°C in PBS with 2% paraformaldehyde until samples were acquired on an LSRII BD flow cytometer using BD FACSDiva software (BD Biosciences). Flow cytometric analyses were performed using FlowJo version 9 (Tree Star). Criteria for evaluable responses were determined as previously described [39].

**Table 3:** Flow Cytometry Panel Used to Determine Activation and Exhaustion in PBMC Samples.

Marker	Dye	Reference
**Viability**		
AViD	V510	[38]
**T cell**		
CD3	BV650	[40]
CD4	APCAx750	[41]
CD8	PerCPCy5.5	[42]
**Activation and Exhaustion**		
CD25	PE594	[43]
CD38	PE-Cy5	[11, 44]
CD127	BV786	[45]
CTLA4	APC	[46]
HLA-DR	BV605	[11]
PD1	BV421	[16]
Ki67	FITC	[47]

**S1 Fig** shows the gating and staining strategy to identify CD4+ and CD8+ cells and activation and exhaustion markers. A timing gate was used to minimize exclusion events, and dead cells were excluded using the AViD (i.e., amine reactive dye) stain. In addition, single cell populations were gated, and within the lymphocyte gate, CD3+ cells were identified followed by gating on CD4+ or CD8+ cells. Using the OMIP-025 panel [48], a subset of HIV+ PBMC samples (N = 4) were separately stained using a T cell differentiation and memory panel. All HIV+ PBMC, in both panels, were stained with LIVE/DEAD® Fixable Aqua Dead Cell Stain Kit (ThermoFisher Scientific, Waltham, MA, USA), washed, and stained with the activation/exhaustion or differentiation/memory staining panels.

### Luminex experiments

Seven chronically-infected, HIV+ PBMC samples were thawed and treated with SM (80 μM) or DMSO (vehicle control), cultured for 72 hours, with supernatants added to a final 1% Triton-X (Sigma-Aldrich, St. Louis, MO, USA) concentration, and then processed for Luminex analysis. The Cytokine Laboratory, a Shared Resource of the Fred Hutchinson Cancer Research Center, processed the Luminex samples.

### Statistical analyses

For MAIT cells and monocyte experiments, differences in cytokine production were assessed by paired Wilcoxon signed rank tests. Twenty-five PBMC samples were included in the final flow cytometry analyses. We measured changes in marker expression on cells with SM treatment relative to DMSO alone, and computed absolute differences in percentages of marker expression (i.e., SM minus DMSO treatments). Subsequently, we assessed non-parametrically whether differences in expected expression tended to be statistically significantly different from zero, using a Wilcoxon signed rank test. Differences in expression with SM treatment minus expression with DMSO-only treatment are negative if SM reduced expression and positive if it increased expression. For markers showing a significant expression reduction with SM (versus DMSO), we also used Spearman’s correlation to assess whether expression reduction was associated with continuous measures like age and CD4:CD8 ratio; and we used Wilcoxon rank sum test to assess whether expression reduction was associated with binary factors like ART use.

## Results

**Fig 1:**
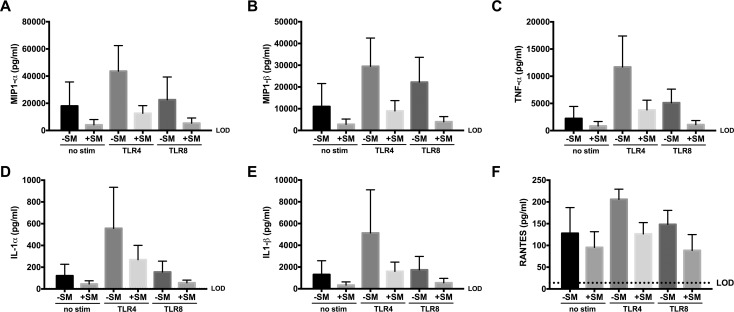
SM inhibits basal and PAMP-induction of pro-inflammatory cytokines from primary human monocytes. Luminex analysis of MIP1α (A), MIP1β (B), TNF-α (C), IL1α (D), IL1β (E), and RANTES (F) from supernatants collected from CD14+ monocytes cultured in the absence or presence of SM (80 μM) for 24 hours in different activating conditions: rested (black), LPS-stimulated, a TLR4 agonist (dark gray), or ssRNA-stimulated, a TLR8 agonist (light gray). Data shown are from three different donors, and data are displayed as mean +/- SEM. Dotted line represents limit of detection (LOD).

We have previously shown that SM prevents activation and inflammatory responses in CD4+ and CD8+ T cells from both non-infected and HCV-infected subjects [21, 22, 32]. Therefore, in the current study, we first investigated the effects of SM on other primary human immune cells from non-infected donors stimulated in the context of different triggers of inflammation. SM treatment showed a trend for reduced levels of MIP1-α, MIP1-β, IL-1α, IL1-β, RANTES, and TNF-α production from both non-stimulated and PAMP-stimulated monocytes (**Fig 1**). Due to the small sample size (i.e., 3 donors), the observed effect did not reach statistical significance.

**Fig 2:**
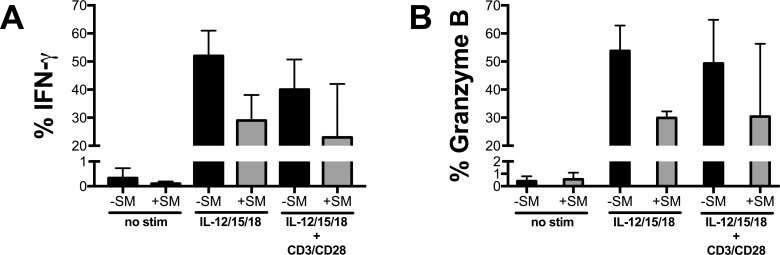
SM suppresses inflammation in resting and cytokine- and TCR-activated MAIT cells. Expression of IFN-γ (A) and granzyme B (B) by CD8+ Va7.2+ CD161hi sorted MAIT cells cultured for 24 hours at rest, 100ng/ml IL-12, 15, and 18, or a combination of IL-12, 15, 18 and anti-CD3/CD28 beads, in the presence or absence of SM (80 μM). Data shown are from three different donors, and data are displayed as mean +/- SEM.

Mucosal-associated invariant T (MAIT) cells have a memory-like phenotype, make up one to eight percent of circulating T cells, and are activated by IL-12, 15, and 18 to produce pro-inflammatory cytokines TNF-α and IFN-γ, and granzyme B [49, 50]. SM treatment also showed a trend for suppressing basal, cytokine (IL-12, 15, and 18), and cytokine and TCR-mediated (IL-12, 15, and 18 plus anti-CD3/28 beads) IFN-γ and Granzyme B in CD8+ MAIT cells (**Fig 2)**. For this small sample size of three donors, the effects did not reach statistical significance. The data suggest that SM treatment suppresses inflammation in resting and PAMP-, cytokine-, and TCR-activated primary human monocyte and CD8^+^ MAIT cell types.

In the current study, we interrogated expression of traditional activation markers of CIA (e.g., HLA-DR and CD38), as well as other markers of T-cell activation (e.g., Ki-67 [30]) and markers for T-cell exhaustion, including PD-1 and CTLA-4 [40] using our flow cytometry panel (**Table 3**). We analyzed the effect of SM treatment on T cell marker expression from 25 human PBMC samples (**Table 2**) including: four HIV-negative subjects, and 21 subjects who were HIV-positive (i.e., 14 subjects were on ART and seven subjects were not on ART). We used a dose of 80μM SM, which we have previously shown to be non-toxic to human cells [31]. We monitored cell viability of all PBMC cultures, determined as the percentage of AViD-negative cells. Using a Wilcoxon paired test, the 80μM dose of SM, after 72 hours of *ex vivo* cell culture, induced a small, but significant (p<0.0001) decrease in viability: the median decrease of viability due to SM treatment relative to DMSO was 7%, (IQR [2%–12%]). Twenty-five PBMC samples had greater than 50% viability and were included in statistical analyses. Moreover, all flow cytometry data described below are based on viable cells.

**Fig 3:**
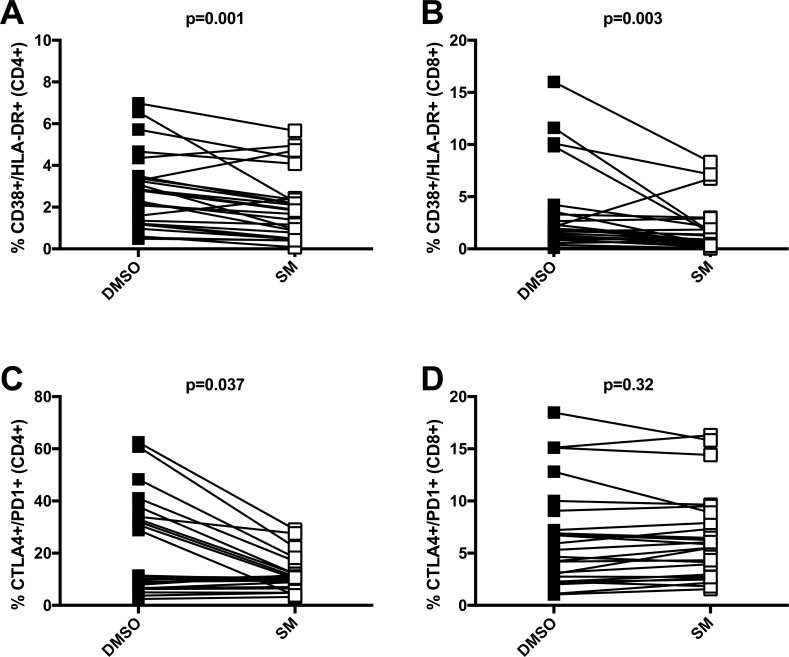
SM suppresses the expression of activation and exhaustion markers on CD4+ and CD8+ T cells from HIV+ subjects. Expression of activation markers, CD38 and HLA-DR on CD4+ (A) and CD8+ (B) T cells, and exhaustion markers, CTLA4 and PD1, on CD4+ (C) and CD8+ (D) T cells from PBMC cultures treated for 72 hours with SM (80 μM; empty symbols) or DMSO (vehicle control; solid symbols). Data are from 25 different PBMC samples. P values are derived from Wilcoxon signed rank tests.

SM treatment reduced the expression of activation markers in HIV-negative and HIV-positive T cells (**Table 4** and **S2 Fig)**. Specifically, SM treatment significantly reduced the expression of CD38+/HLA-DR+ (compared to DMSO) in both CD4+ and CD8+ T cells (i.e., difference of -0.7%; median IQR [-1.1% to -0.4%]; p-value = 0.001) and (-0.6%; [-1.7% to -0.3%]; p-value = 0.0003), respectively (**Fig 3**). SM caused a greater suppression of CD38+/HLA-DR+ expression on T cells from ART-naïve patients as compared to patients on ART. This effect was observed on both CD4+ (p-value = 0.001) and CD8+ (p-value = 0.0074) T cells **(S3A–S3D Fig)**.

**Table 4:** SM Suppresses T Cell Activation and Exhaustion Markers.

Marker	CD4 T cells		CD8 T cells	
	SM Effect*	p-value**	SM Effect*	p-value**
CD38+/HLA-DR+	-0.7% (-1.1% to -0.4%)	0.001	-0.6% (-1.7% to -0.3%)	0.0003
CD25+/CD127lo	-0.7% (-1.6% to 1.6%)	0.71	0.0% (-0.0% to 1.0%)	0.046
Ki67+	0.01% (-0.03% to 0.04%)	0.92	-0.01% (-0.04% to 0.00%)	0.085
CTLA4+/ PD1+	-0.9% (-21.9% to 0.5%)	0.037	0.4% (-0.5% to 0.8%)	0.32

*SM Effect represents the median differences in percentages of marker expression (i.e., SM minus DMSO treatments). The bracketed values show the interquartile range.

** Wilcoxon signed rank test for a difference of paired data.

In the CD4+ T cell population, the median difference in CD38+/HLA-DR+ expression between SM and DMSO treated cells was -1.3% (IQR -1.8% to -1.1%) for ART-naïve individuals and was -0.5% (IQR -0.7% to -0.2%) for subjects on ART (**S2A and S2B Fig**). In the CD8+ T cell population, the median difference of CD38+ and HLA-DR+ expression between SM and DMSO treated cells was -3.3% (-8.0% to -0.9%) for ART-naïve individuals and was 0.4% (IQR -0.8% to 0.1%) for subjects on ART (**S2C and S2D Fig**). Lower CD4:CD8 ratio was associated with a significant decrease in CD38+ and HLA-DR+ expression on SM versus DMSO treated CD8+ T cells (Spearman’s rho = 0.65, p-value = 0.002; **S2E and S2F Fig**) but was not significantly associated with a decrease on CD4+ T cells (Spearman’s rho = 0.37, p = 0.095). There were no age-related associations in the difference of CD38+/HLA-DR+ expression for either CD4+ or CD8+ T cells (p = 0.27 and p = 0.34, respectively).

SM treatment also suppressed the expression of T cell exhaustion markers, CTLA4 and PD1 (**Table 4** and **S3 Fig**). Specifically, CTLA4+/PD1+ expression on CD4+ T cells was significantly reduced by SM treatment in comparison to DMSO (difference of -0.9%; IQR [21.9% to 0.5%]; p-value = 0.037; **Table 4**, **Fig 3**). These effects were limited to CD4+ T cells, as SM did not significantly reduce these markers on CD8+ T cells (p = 0.32). Moreover, the reduction of CTLA4+/PD1+ on CD4+ T cells by SM treatment was strongly associated with CD4:CD8 ratio (Spearman’s rho = 0.57; p-value = 0.010; **S3A and S3B Fig**). As with CD38+/HLA-DR+ markers, no association of CTLA4+/PD1+ markers was observed with age (p = 0.79). However, SM reduction of CTLA4+/PD1+ markers on CD4+ T cells was greater in magnitude in PBMC samples from individuals that were ART-naïve (median difference of -24.2%; IQR [-31.8–21.0%]) versus those that were receiving ART (-0.1% [IQR -6.4% to 0.4%], Wilcoxon test, p = 0.037) (**S3C and S3D Fig**).

After 72 hours in culture, SM did not significantly alter the expression of the activation marker Ki67 in CD4+ and CD8+ T cells (**Table 4; S4A and S4B Fig**). In addition, SM treatment did not have any significant effect on CD4+ regulatory T cell markers (i.e., Tregs; defined as CD25+/CD127lo CD4+ T cells; p-value = 0.71), while SM treatment marginally reduced the expression of CD25+/CD127lo on CD8+ T cells (p-value = 0.046) (**Table 4; S4C and S4D Fig**).

To determine if SM treatment results in the inhibition of T cell pro-inflammatory function, a subset of chronically infected, HIV+ PBMC culture supernatants were harvested after 72 hours of culture in SM or DMSO and processed for Luminex analyses. As shown in **S5 Fig**, SM treatment suppressed IL-18 and IL-6 expression in cultures, whereas the effect of SM on IL-8 expression was variable.

**Fig 4:**
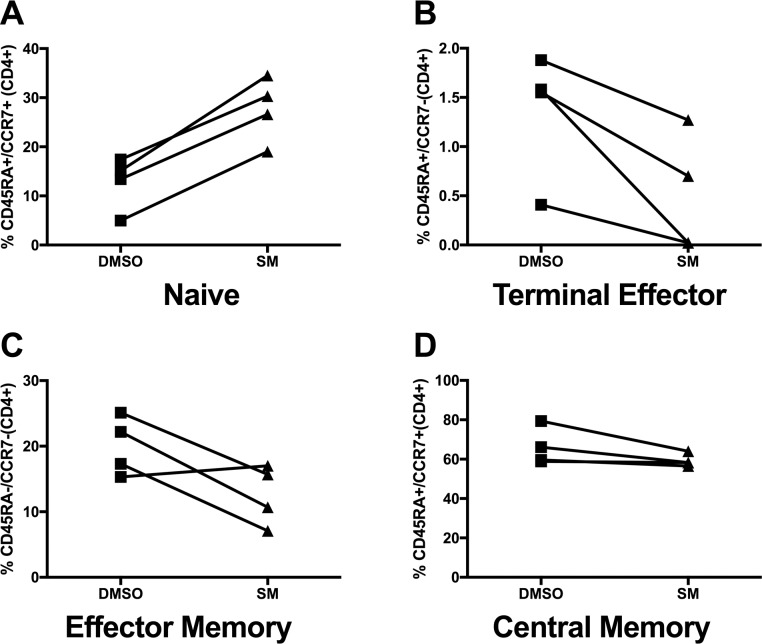
SM affects the differentiation states of CD4+ T cell populations in HIV+ PBMC cultures. After 72 hours of treatment with SM (80 μM; empty symbols) or DMSO (vehicle control; solid symbols), PBMC were analyzed by flow cytometry for CD4+ T cell differentiation markers, defined as follows: A, Naïve (CD45RA+/CCR7+), B, Terminal Effector (CD45RA+/CCR7-), C, Effector Memory (CD45RA-/CCR7-), and D, Central Memory (CD45RA-/CCR7+).

The chemokine receptor CCR7 and the tyrosine phosphatase CD45RA are used to designate T cells as naïve (CD45RA+/CCR7+), terminal effector (CD45RA+/CCR7-), central memory (CD45RA-/CCR7+), and effector memory (CD45RA-/CCR7-) cells [51, 52]. While not statistically significant due to the low number of samples (N = 4), SM treatment showed a trend for increasing the percentage of naïve CD4+ T cells (defined as CD4+ cells expressing CD45RA+/CCR7+ markers) in comparison to DMSO treatment (**Fig 4**). SM treatment also showed a trend towards reducing the percentage of CD4 T cells in the terminal effector and effector memory cell subsets.

## Discussion

In this study, we show that SM significantly reduces expression of various markers of T cell activation and exhaustion (e.g., CD38, HLA-DR, CTLA4, and PD1) as well as pro-inflammatory cytokines (e.g., IL-6 and IL-18) in PBMCs from chronically-infected HIV-positive subjects. SM treatment also showed a trend towards maintaining naïve CD4+ T cell populations in cultured PBMC from chronically infected, HIV+ subjects. For monocytes and MAIT cells in the HIV-negative setting, SM treatment showed trends toward reducing pro-inflammatory cytokines in resting/non-stimulated, and cytokine-, PAMP-, and TCR-activated cultures. While interesting, these effects of SM on monocytes, MAIT cells, and T cell memory subsets need to be bolstered in future studies by studying larger numbers of samples. Nevertheless, the data indicate that the inflammatory status of primary human immune cells, in both resting and stimulus-activated conditions, is suppressed by SM.

Similar to activation and exhaustion markers, SM suppression of PAMP-induced inflammation in monocytes may be important during CIA. For example, monocyte activation has been associated with systemic inflammation, atherosclerosis, and the development of premature cardiovascular disease [53], all of which occur at a higher rate in the HIV-infected community [54, 55].

The effects of SM treatment may not only be applicable to monocytes and T cells in the context of HIV infection, as SM treatment may also be effective for the suppression of MAIT activation involved in resetting pathologic inflammation. Multiple studies have shown that CD8+ MAITs contribute to the primary, innate-like response against bacterial and fungal infections and can be activated by inflammatory stimuli [36, 56]. The suppression of CD8+ MAIT cell activation, as shown here by SM treatment, is not limited to that caused by acute bacterial infections, but may also be important during chronic viral infections, such as HIV: circulating MAIT cells are severely reduced in chronically-infected HIV+ patients, and fail to completely restore to pre-infection frequencies with ART [57]. Moreover, these MAIT cells showed a functionally exhausted phenotype. Our data suggest that by treating MAIT cells with SM and reducing their inflammatory response, it may be possible to reduce the MAIT-associated exhausted phenotype, preserving their function in both the blood and at epithelial sites, such as in the context of chronic viral infections.

The reduction of the T cell activation markers CD38 and HLA-DR by SM was correlated with a low CD4:CD8 ratio (i.e., < 1) and in PBMC from subjects not on ART. In the setting of chronic HIV infection, clinical parameters such as CD4:CD8 ratio have been used to identify HIV+ individuals on ART who are at greater risk of immune dysfunction, leading to AIDS, non-AIDS events, and mortality [58]. Thus, natural products like SM may be effective in reducing CIA that is associated with AIDS, non-AIDS events, and mortality. In addition to the traditional activation markers CD38+/HLA-DR+, the reduction of exhaustion markers CTLA4 and PD1 by SM may also be clinically relevant in the context of HIV-associated CIA. For example, blocking PD-1 receptor function during simian immunodeficiency virus *in vivo* was shown to improve virus-specific CD8+ T cell responses, reduce plasma viremia, as well as increase animal survival [59]. In fact, by inhibiting multiple immune exhaustion pathways (e.g., PD-1 and CTLA4) simultaneously, T cell function can further be improved [60], suggesting potential additive or synergistic therapies for recovery of immune function. In the context of chronic HIV infection, the ability of SM to reduce CTLA4 and PD1 expression is particularly interesting, since cells with high-level expression of exhaustion markers may preferentially harbor latent, HIV proviral DNA [61].

The ability of SM to quell various types of inflammation is similar to the immunomodulatory effects of metabolic modulators like rapamycin, which can suppress cellular activation, stimulus-induced inflammation, and alter immune cell fate (i.e. differentiation). Rapamycin’s principle mechanism of action is via inhibition of mammalian target of rapamycin (mTOR) through its association with FKBP12 [62, 63]. We have recently shown that SM also inhibits mTOR by at least two pathways, involving activation of adenosine monophosphate kinase (AMPK) and induction of DNA Damage Inducible Transcript 4 (DDIT4), a novel inhibitor of mTOR [31]. Moreover, SM activates AMPK signaling, which feeds forward to suppress NF-κB signaling [31]. Thus, SM appears to modulate multiple metabolic pathways that converge on suppressing inflammation in diverse immune cell types, independent of the inflammatory insult or stimulus.

## Supporting information

S1 Fig Example of gating tree used for flow cytometry data. A representative example of a PBMC sample, cultured with DMSO (vehicle control) for 72 hours, stained with the immune exhaustion panel. Gating is as follows: *top row*, Time, Singlet, AViD Live/Dead. *Second row*, Lymphocyte, CD3+, CD4+ and CD8+ Cells. *Third and forth rows*, CD4+ and CD8+ cells, respectively, gated for CD25hi/CD127lo, CD38+/HLA-DR+, Ki67hi, and CTLA4+/PD1+.
(PDF)

S2 Fig Relationship of SM Suppression of CD38+/HLA-DR+ in CD4+ and CD8+ T cells with ART status and CD4:CD8 ratio. Panels A and B, correlations of SM suppression of activation markers CD38 and HLA-DR on CD4+ T cells with ART status. Panel A is the actual data while panel B plots the difference. Panels C and D, correlations of SM suppression of activation markers CD38 and HLA-DR on CD8+ T cells with ART status. Panel C is the actual data while panel D plots the difference. Panels E and F, association of SM suppression of CD38 and HLA-DR on CD8+ T cells with CD4:CD8 ratio.
(PDF)

S3 Fig Suppression of CTLA4/PD1 on CD4+ T cells is associated with CD4:CD8 ratio and ART status. Panels A and B, difference in CTLA/PD1 expression was strongly associated with CD4:8 ratio for CD4+ T cells. Panels C and D, SM reduction of CTLA/PD1 on CD4+ T cells was greater in samples from ART-naïve individuals.
(PDF)

S4 Fig SM does not alter the expression of activation (Ki67) and T regulatory cell (Treg) markers on PBMC from HIV-infected individuals. PBMC were thawed and cultured for 72 hours in either SM (80 μM; empty symbols) or DMSO (vehicle control; solid symbols) followed by staining with the exhaustion panel (listed in Table 3). Samples were assessed for the activation marker Ki67 on CD4+ (A) and CD8+ (B) T cells, and Treg markers, defined as expression of CD25+/CD127lo on CD4+ (C) and CD8+ (D) T cells.
(PDF)

S5 Fig SM suppresses pro-inflammatory cytokines in HIV+ PBMC cultures. PBMC were thawed and treated with SM (80 μM) or DMSO (vehicle control), cultured for 72 hours, with supernatants lysed by the addition of a final 1% Triton-X concentration, and then processed for Luminex analysis. Data shown are from seven different PBMC samples.
(PDF)

## Abbreviations

AMPK: adenosine monophosphate kinase
ART: antiretroviral therapy
CIA: chronic immune activation
DMSO: dimethyl sulfoxide
FACS: fluorescence-activated cell sorting
HCV: hepatitis C virus
HIV: human immunodeficiency virus
IQR: interquartile range
MAIT: mucosal associated invariant T
mTOR: mammalian target of rapamycin
PAMP: pathogen-associated molecular pattern
PBMC: peripheral blood mononuclear cell
SM: silymarin
TCR: T cell receptor
