# Supplementary figures and images for: Silymarin suppresses basal and stimulus-induced activation, exhaustion, differentiation, and inflammatory markers in primary human immune cells
Source: PLoS One. 2017 Feb 3;12(2):e0171139. doi: 10.1371/journal.pone.0171139 (PMC5291532; doi:10.1371/journal.pone.0171139)

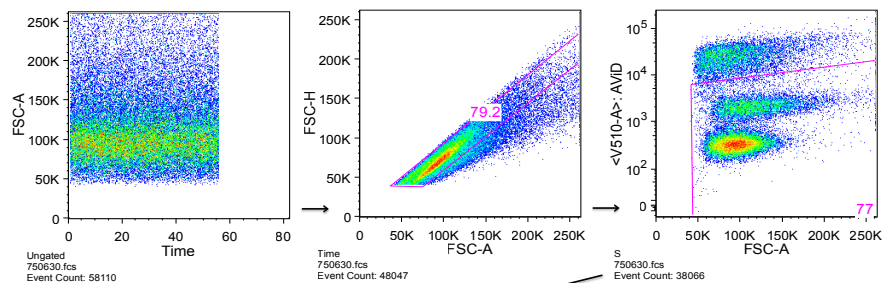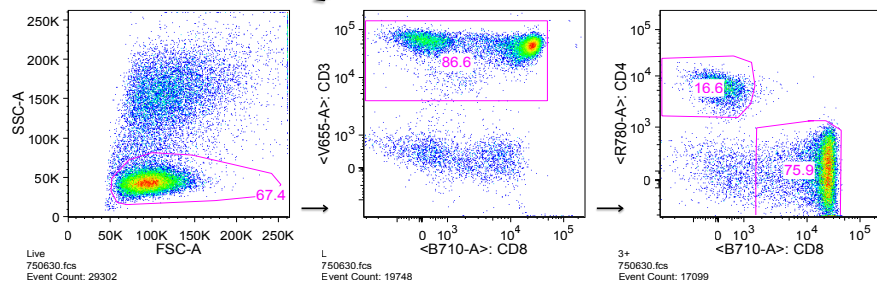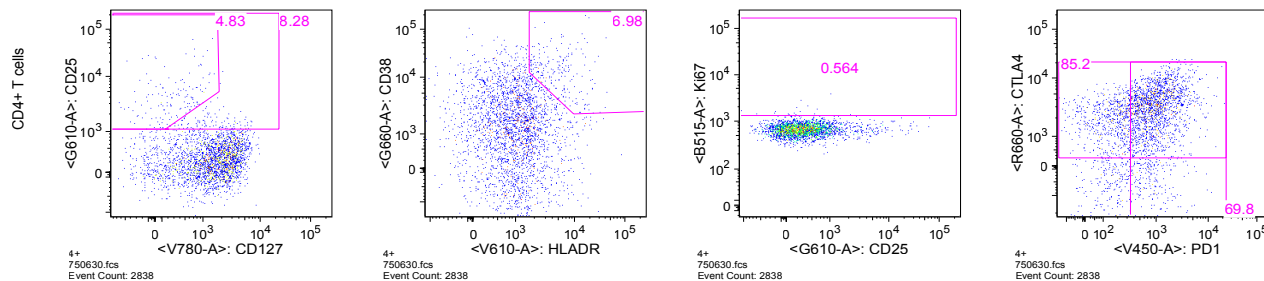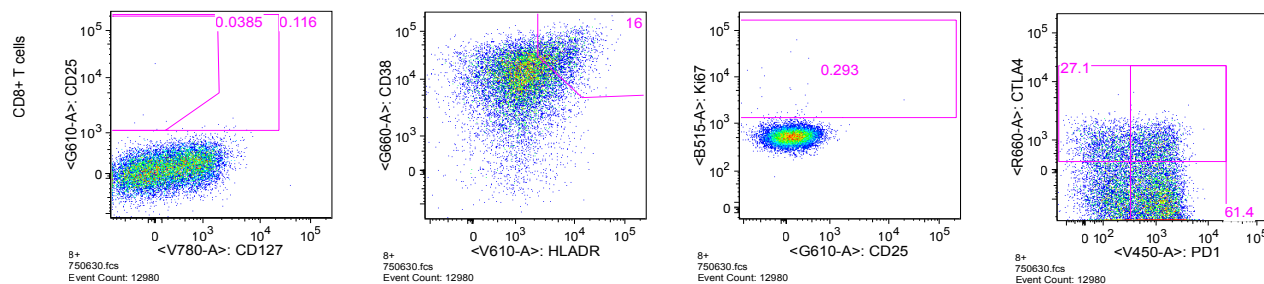

Supplement: S1 Fig — A representative example of a PBMC sample, cultured with DMSO (vehicle control) for 72 hours, stained with the immune exhaustion panel. Gating is as follows: top row, Time, Singlet, AViD Live/Dead. Second row, Lymphocyte, CD3+, CD4+ and CD8+ Cells. Third and forth rows, CD4+ and CD8+ cells, respectively, gated for CD25hi/CD127lo, CD38+/HLA-DR+, Ki67hi, and CTLA4+/PD1+. (PDF) [file pone.0171139.s001.pdf]

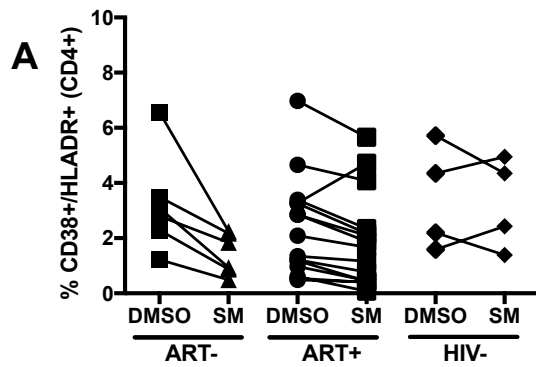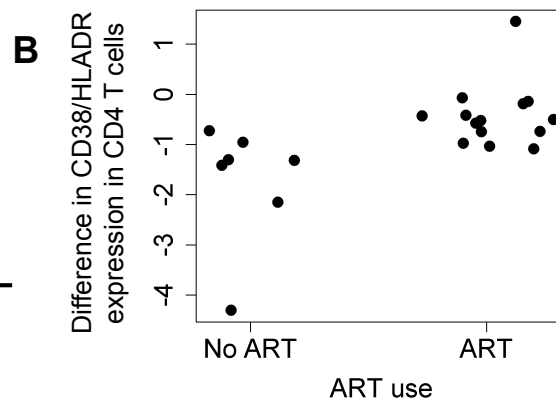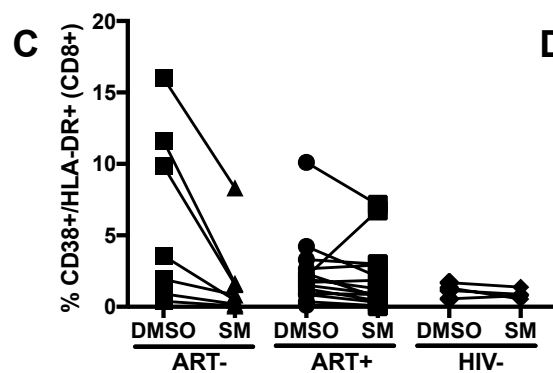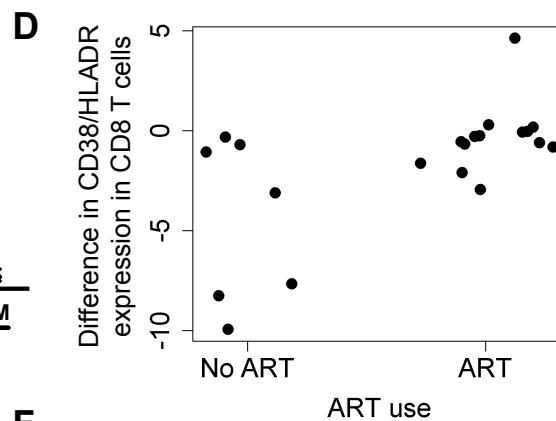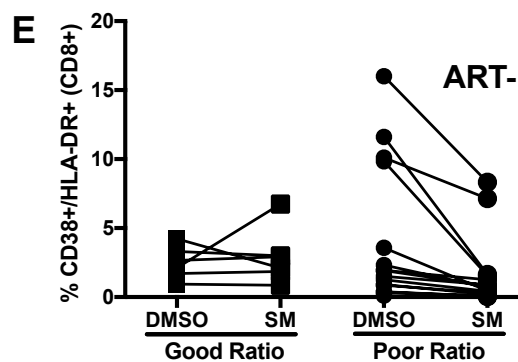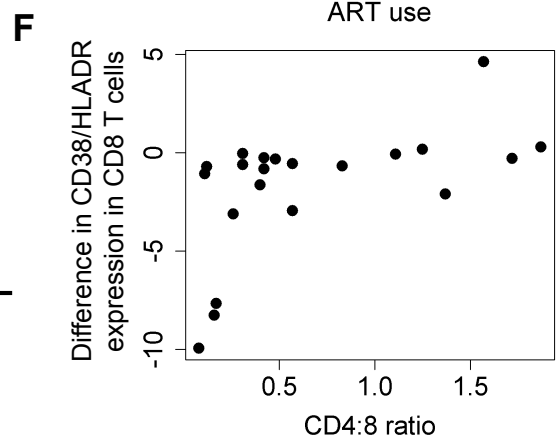

Supplement: S2 Fig — Panels A and B, correlations of SM suppression of activation markers CD38 and HLA-DR on CD4+ T cells with ART status. Panel A is the actual data while panel B plots the difference. Panels C and D, correlations of SM suppression of activation markers CD38 and HLA-DR on CD8+ T cells with ART status. Panel C is the actual data while panel D plots the difference. Panels E and F, association of SM suppression of CD38 and HLA-DR on CD8+ T cells with CD4:CD8 ratio. (PDF) [file pone.0171139.s002.pdf]

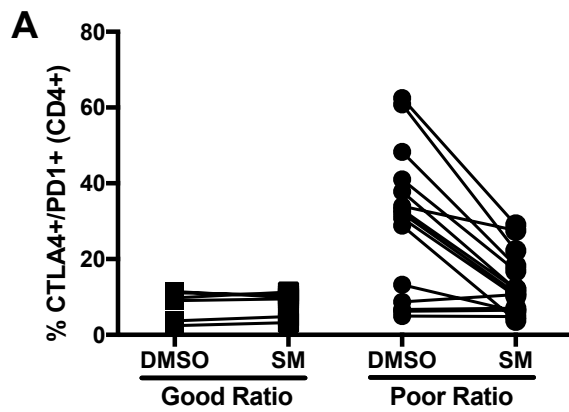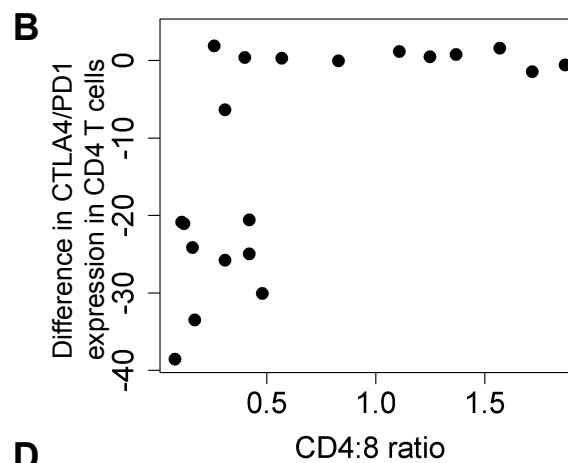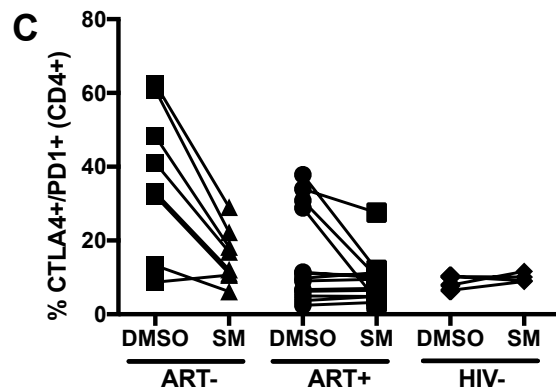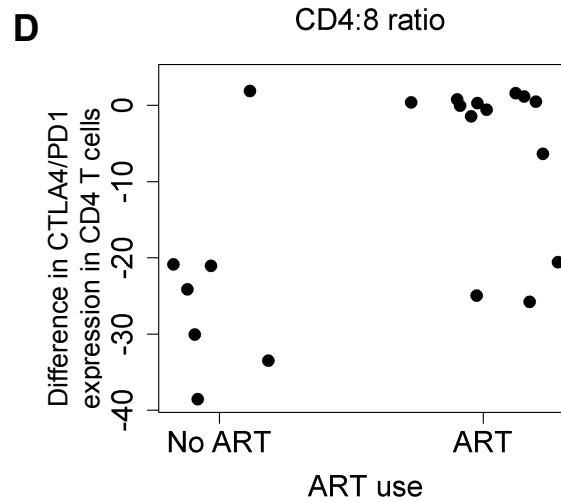

Supplement: S3 Fig — Panels A and B, difference in CTLA/PD1 expression was strongly associated with CD4:8 ratio for CD4+ T cells. Panels C and D, SM reduction of CTLA/PD1 on CD4+ T cells was greater in samples from ART-naïve individuals. (PDF) [file pone.0171139.s003.pdf]

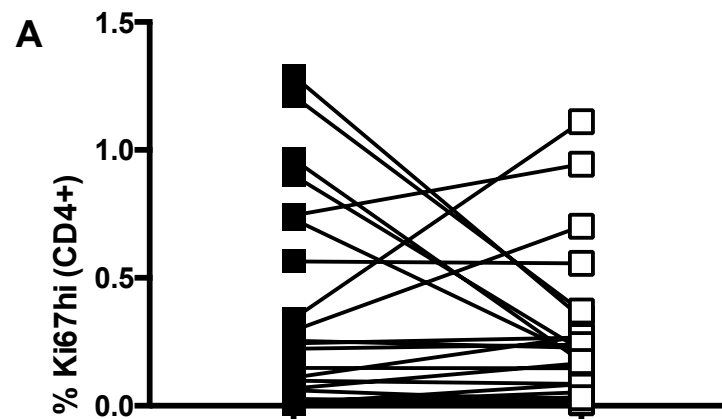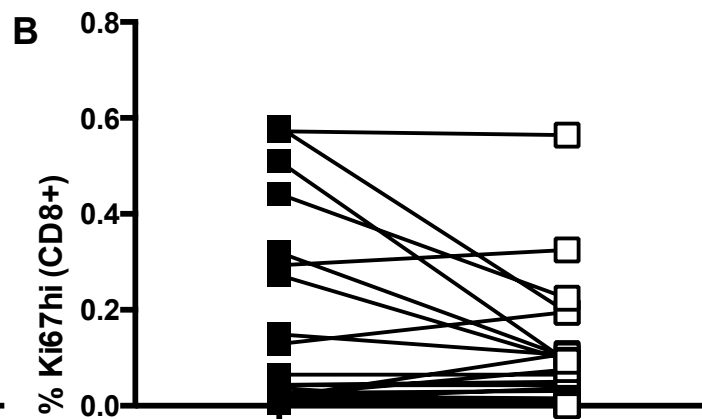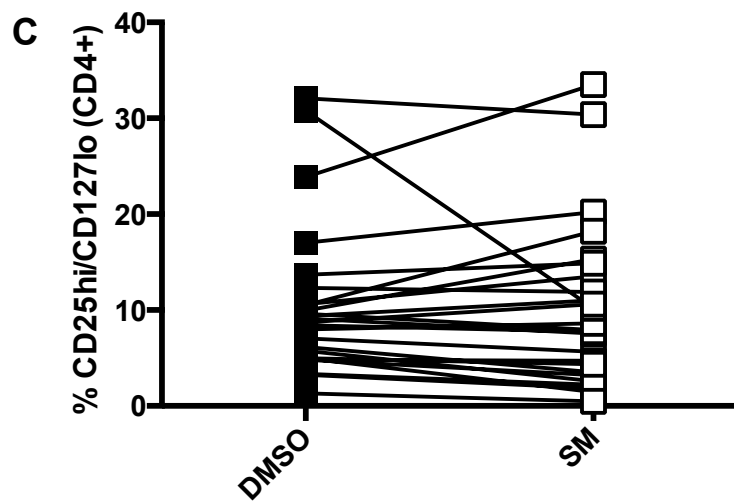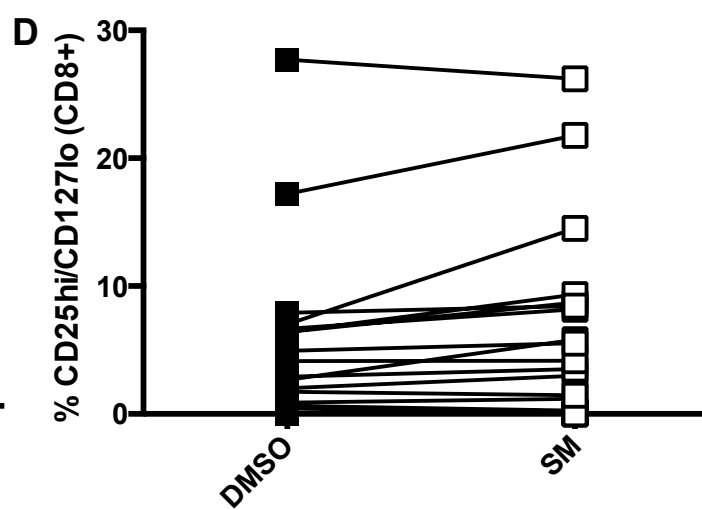

Supplement: S4 Fig — PBMC were thawed and cultured for 72 hours in either SM (80 μM; empty symbols) or DMSO (vehicle control; solid symbols) followed by staining with the exhaustion panel (listed in Table 3). Samples were assessed for the activation marker Ki67 on CD4+ (A) and CD8+ (B) T cells, and Treg markers, defined as expression of CD25+/CD127lo on CD4+ (C) and CD8+ (D) T cells. (PDF) [file pone.0171139.s004.pdf]

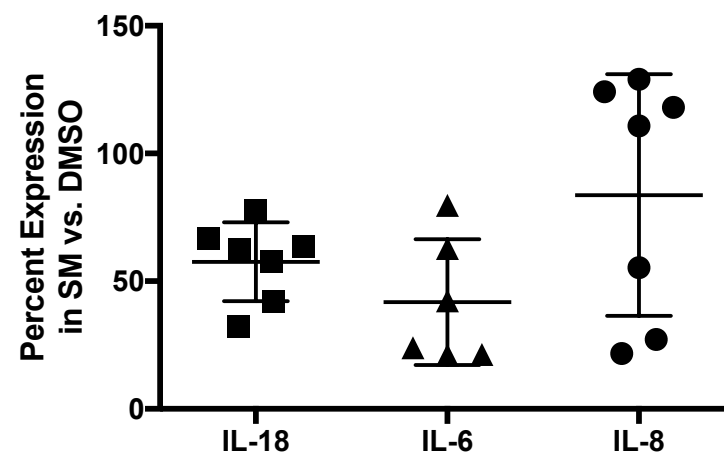

Supplement: S5 Fig — PBMC were thawed and treated with SM (80 μM) or DMSO (vehicle control), cultured for 72 hours, with supernatants lysed by the addition of a final 1% Triton-X concentration, and then processed for Luminex analysis. Data shown are from seven different PBMC samples. (PDF) [file pone.0171139.s005.pdf]
